# Thermo-chemical conversion kinetics of cannabinoid acids in hemp (*Cannabis sativa L*.) using pressurized liquid extraction

**DOI:** 10.1186/s42238-024-00243-x

**Published:** 2024-07-30

**Authors:** Urvashi, Joon-Hee Han, Min Hong, Tae-Hyung Kwon, Melvin Druelinger, Sang-Hyuck Park, Chad A. Kinney, Kenneth J. Olejar

**Affiliations:** 1 Chemistry Department, Colorado State State University – Pueblo, 2200 Bonforte Blvd, Pueblo, Colorado 81001-4901 USA; 2 Institute of Biological Resources, CBF, Chuncheon Bioindustry Foundation, Chuncheon, 24232 Republic of Korea; 3 Institute of Cannabis Research, Colorado State State University – Pueblo, Pueblo, Colorado 81001 USA

**Keywords:** Thermo-chemical conversion, Pressurized liquid extraction, Cannabinoids, Decarboxylation, Reaction kinetics

## Abstract

Cannabinoid decarboxylation via thermo-chemical conversion has the potential to reduce the cannabinoid degradation and evaporation due to short reaction time and use of water as the solvent. When combined with pressurized liquid extraction (PLE), thermo-chemical conversion can be performed as the first stage in the extraction procedure. PLE utilizes a closed system at elevated temperatures and pressure to increase the solvation power, which contributes to decreased viscosity and increased diffusion rate. With this new *in-extraction* decarboxylation approach there remain variables that need full understanding before up scaling from bench top to pilot or commercial scale. Herein, the thermo-chemical decarboxylation kinetics was studied for industrial hemp via PLE at different temperatures (80–160 °C) and reaction times (1–90 min). The reaction was found to be pseudo-first order. Model verification on CBD and CBG resulted in acceptable results; however, an anomaly in the minor cannabinoids suggests that cannabinoid concentration may influence model kinetics.

## Background

*Cannabis sativa* is a plant that has been widely used for recreational and medicinal purposes for millennia. Recently, cannabis has become of increased interest amongst the scientific community, after its legal status change brought about by the 2014 Farm Bill, allowing research at institutions of higher education in states that have regulatory frame work for cannabis (U.S. Department of Agriculture, n.d.) and interest in its potential to treat the conditions such as epileptic seizures, sclerosis, chemotherapy caused nausea, pain, and anxiety (Hao et al., 2015; Russo & Marcu, 2017; Hartsel et al., 2016). Cannabinoids or isoprenylated resorcinyl polyketides are biosynthesized and stored in the glandular trichomes of flower bracts (Desaulniers Brousseau et al., 2021; Christelle et al., 2016) and primarily exist as their carboxylic acids. Out of more than 100 cannabinoids, only a handful have been researched in extensive detail, e.g., cannabidiol (CBD) and Δ^9^-tetrahydrocannabinol (THC), as they are major cannabinoids between numerous chemotypes. Cannabis varieties, holding low amounts of THC are commonly termed as industrial hemp and are produced for their higher production of other cannabinoids or fiber production (Olejar et al., 2021). In Colorado, where this study took place, industrial hemp is any cannabis variety containing < 0.3% THC_total_, similar to the definition in the European Union.

The neutral forms of cannabinoids do not occur in significant amounts in plant biomass, but they are obtained after applying a decarboxylation process (e.g., heat, light and chemical methods) on naturally available acidic cannabinoids. The decarboxylation process transforms raw acidic cannabinoids into their biologically active and stable form, based on proper storage in appropriate solvent. These neutral forms interact with the endocannabinoid system (ECS), a network of cannabinoid receptors (i.e., CB1 and CB2) found throughout the body including the central and peripheral nervous system (Russo & Marcu, 2017). A varying effect of euphoria to relaxation can also be seen as the result of a few major and minor cannabinoids.

In this non-enzymatic decarboxylative process, the -COOH group is lost as carbon dioxide (CO_2_), while retaining one hydrogen atom. Several environmental factors, such as temperatures, light, and oxygen can affect this conversion (Hartsel et al., 2016) and this process results in a significant concentration of the neutral cannabinoids. Traditionally, decarboxylation is done by applying heat higher than 120 °C temperature, as in the process of smoking, vaping, and baking (Lewis-Bakker et al., 2019; Rochfort et al., 2020). However, for better efficiency a more controlled approach would be beneficial.

Decarboxylation can be performed either before or after extraction of crude oil, based on solvent polarity used in the method. In the case of polar solvent extraction, decarboxylation is generally carried out on the extracted oil, as only small volumes are required. On the other hand, in the non-polar extraction case, decarboxylation is often applied before extraction of the crude oil. The latter has the advantage of removing residual moisture from the plant material thereby making the cannabinoids more soluble in the extraction solvent (Moreno et al., 2020). An alternative option, thermo-chemical conversion, utilizes water as a solvent under elevated pressure and temperature to decarboxylate the acidic cannabinoids. This process is appealing as water is inexpensive, environmentally benign, non-flammable, non-toxic, and thermo-chemical conversion has been shown to minimize loss due to degradation (Olejar et al., 2021; Olejar & Kinney, 2021). Moreover, with globalization and cannabis becoming accepted as a source of medication, and to minimize the chance of product variation for this application, the cannabis manufacturer must adhere to current good manufacturing procedures (cGMP) and other regulatory guidelines, which limits the amounts of solvents that can be present in the end product.

Early attempts to maximize extraction started with the quantitative measurement of THC by Kimura & Okamoto (1967), using decarboxylation at 100 °C for 15 min, followed by Kanter et al. (1979) who utilized 200 °C for 3 min. Subsequently, Veress et al. (1990) investigated the decarboxylation kinetics of THC using solvent extraction of dried flowers in open reactors and ultimately concluded it to be a first order reaction.

**Fig. 1:**
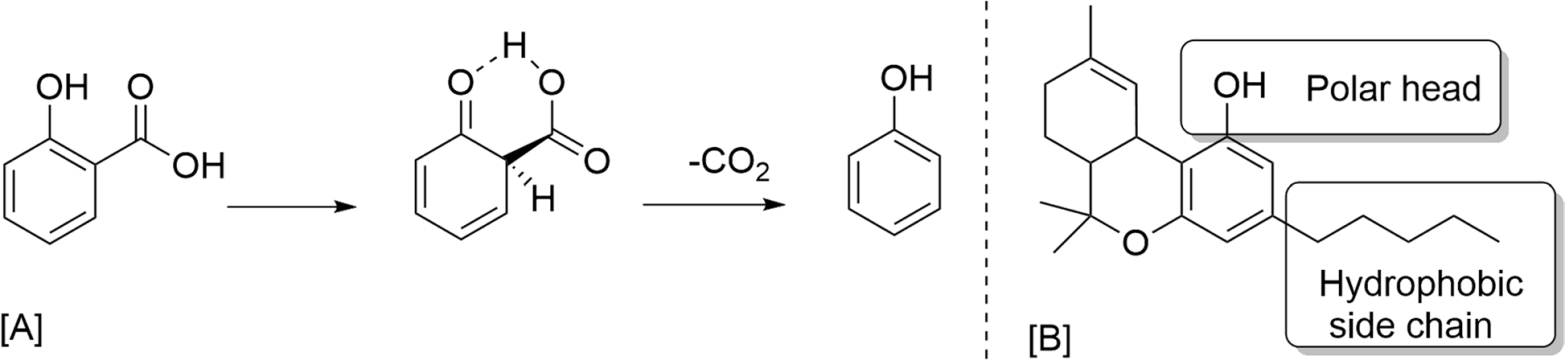
**A** Decarboxylation through β-keto acid mechanism in 2-hydroxybenzoic acid; (**B**) structure of Δ^9^-THC cannabinoid

Following the 2018 US Farm Bill, studies changed from analytical analysis methods to focus on production methods. Perrotin-Brunel et al. (2011) published one of the first studies in this new environment that expressed two possible routes, namely direct and indirect decarboxylation, for an acid catalyzed *β*-keto acid type mechanism (Fig. 1) for Δ^9^-tetrahydrocannabinolic acid (THCA) decarboxylation. Each of the proposed routes might be effective based on the actual process conditions. The study carried out with a small amount of cannabis over a range of temperatures (90—140 °C). To monitor the reaction rate, samples were taken periodically until they were completely decarboxylated, resulting in a pseudo-first order reaction with the variation of the rate constant depending on temperature change. Molecular modeling concluded that the formic acid present as a chain organic acid in the cannabis flowers catalyzed the decarboxylation reaction.

A qualitative HPLC–UV based determination of THCA, cannabigerolic acid (CBGA), and cannabidiolic acid (CBDA) decarboxylation was examined by Citti et al. (2018). Using both open and closed reactors for the decarboxylation of 13 commercial hemp seed oils, it was demonstrated that the decarboxylation processes were first order reactions. A similar set of experiments for major cannabinoids decarboxylation (THCA, CBDA and CBGA) establishing a first order or pseudo first order reaction was carried out by Wang et al. (2016) using cannabis extracts in a vacuum oven. At different temperatures, the conversion showed an exponential behavior between time and concentration. The rate constant for CBDA and CBGA observed in cannabis extracts was nearly half of the rate constant of THCA. The mass balance in THCA to THC reaction was forthright with 1:1 stoichiometric conversion, indicating no side reactions or cannabinol (CBN) formation (a byproduct after oxidation). Contrariwise, a complicated decarboxylative chemistry for CBDA and CBGA was observed with a loss of 18% and 53% respectively, including unexplained side reactions. Further degradation in CBD might not be the sole reason for this loss, since CBD has shown greater stability over THC, at room temperature (20–22 °C) with light exposure (Lindholst, 2010; Trofin et al., 2012). Zaharia et al. (2020) has also shown the influence of temperature and time on THCA and CBDA decarboxylation in cannabis inflorescences. The report mentioned that the rate constant is directly proportional to the increase in temperature, particularly in the range of 75—150 °C and 100—175 °C for the THCA and CBDA, respectively. Almost constant THC values were recorded throughout all the temperature range, showing complete conversion, although no clear tendency was obtained for the variation of CBD values in accordance with temperatures. This might be due to the secondary side product formation during the transformation. An extensive kinetics study involving several variables (temperature, time, oxygen, and sample mass) for THCA, CBDA and CBGA decarboxylation was reported by Moreno et al. (2020). The order of the reaction agreed with previous literature, nevertheless the mass balance for THCA/THC combining with CBN decreased during the operation, suggesting decarboxylation of THCA was not a 1:1 stoichiometric reaction as reported by Wang et al. (2016). The rates of decarboxylation were found to be directly and indirectly proportional to the presence of oxygen and plant mass correspondingly.

Advance techniques such as pressurized liquid extraction (PLE) (Pavlovic et al., 2019; Montesano et al., 2015; Montesano et al., 2016) and pressurized hot water extraction (PHWE) (Nuapia et al., 2020; Plaza & Turner, 2015) are resulting in significant advantages, such as less degradation, elimination of additional sample clean up, reduction in organic solvent consumption, concentration steps before chromatographic analysis, selectivity, improvement in kinetics, extraction efficiency, and ease of automation, over other methods (Plaza & Turner, 2015). Expeditiously, PHWE maintains water in the supercritical fluid form at high pressure and temperature for the recovery of polar and semi-polar bioactive compounds from plant materials. This method has been widely used for phenolic compounds, with temperature being an important factor that can affect the efficiency and mass transfer during processing (Mohd Jusoh et al., 2019). A recent report on a PHWE system for the optimal conditions to get more CBD, CBC and CBG content than THC and CBN (reducing psycho-activity) was reported for *Cannabis sativa* seed (Nuapia et al., 2020).

Whereas in PLE technique, the insoluble matrix components (in extraction solvent), remain inside the sample extraction cell, hence, not requiring the filtration step. The use of high pressure allows the use of solvent at a temperature above its normal boiling point. This is helpful in extracting analytes efficiently and quickly from various matrices. PLE is advantageous for pairing extraction and separation step together to determine various organic compounds from plant materials and is routinely used, for example in human pharmaceuticals and personal care products (PPCPs) and phytochemicals (Holling et al., 2012; Šulniutė et al., 2017). Unfortunately, Wianowska et al. (2015) showed the transformation of THCA to THC and CBN during decarboxylation in PLE, albeit on a smaller scale. Serna-Loaiza et al. (2020) showed low pressure (50 bar) and 60 min are sufficient to achieve 99.3% efficiency for the extraction of CBD using ethanol. Recently our group, utilizing hemp biomass and PLE, developed a thermo-chemical conversion process, which is capable of decarboxylating cannabinoids (Olejar et al., 2021; Olejar & Kinney, 2021). It was shown that the PLE system utilizing high temperature up to 200 °C and pressure > 10 MPa can effectively decarboxylate and extract the target compounds using less time and solvent (Moreno et al., 2020; Nuapia et al., 2020). This process has demonstrated minimal loss of cannabinoids during the process due to the short time required and the use of water as the solvent during decarboxylation. Additionally, thermo-chemical conversion can be done as an initial step during the extraction process when using PLE. With this new development in decarboxylation, there are variables that remain to be fully understood in order to fully understand the processes at play. One of these variables is the kinetics of the reaction, which will allow for the development of a model that can make the up-scaling process easier as well as provide insights into optimum conditions.

From the kinetics studies the reaction rate constants and activation energies required for the conversion process, as well as those necessary for side reactions and degradation can be determined. Herein, the research describes the determination of the reaction kinetics for thermo-chemical conversion of industrial hemp using PLE as a means of decarboxylation. Furthermore, a model is generated and proposed to reflect the reaction for cannabinoids of interest.

## Methods

### Materials

Industrial hemp biomass consisting of leaf, seeds, stalk, and small inflorescences was obtained from JJN Farms (Trinidad, CO, USA). The varietal used in all the experiments was the hybrid Boax, which originates from *Cannabis sativa* and *Cannabis indica* parents. The varietal is derived from *Cannabis sativa ssp. sativa* Hindu Kush and *Cannabis sativa ssp. indica* Otto II strains. The Boax cultivator is a variety that can provide inflorescences containing 18–20% CBDA and 0–1% THC. This variety was chosen based on consumer market interest in CBD and CBD related products, as well as the varietal availability. The plants were grown in a south to north orientation, outdoors in Colorado. Plant rows were 1.2 m apart and plant spacing was at 0.2 m. Crop management utilized the farm’s management protocol for herbicide, pesticide, and other crop activities. The harvest date was determined by industry practices. The seeds and stalk were removed from the biomass by passing it through sieves. The remaining material consisting of flower and leaf material was then ground and passed over wire mesh until it was able to go through a 1.18 mm screen. The biomass was then stored at 4 °C in sealed bags with air removed to minimize variation between experiments.

### Chemicals

The high-performance liquid chromatography (HPLC) standard solutions of 14 cannabinoids, each at 1 mg/mL in methanol (MeOH) or acetonitrile (Cerilliant, San Antonio, TX, USA), namely cannabichromene (CBC), cannabichromenic acid (CBCA), CBD, CBDA, CBG, CBGA, CBN, cannabidivarin (CBDV), cannabidivarinic acid (CBDVA), THC, Δ^8^- tetrahydrocannabinol (Δ^8^-THC), THCA, tetrahydrocannabivarin (THCV), and tetrahydrocannabivarinic acid (THCVA), were purchased. Both of the stock solutions, all 8 neutral and 6 acidic cannabinoids respectively, each at 100 µg/mL concentration were made in HPLC grade MeOH obtained from Thermo Fisher Scientific (Waltham, MA, USA). A commercial standard solution consisting of 11 cannabinoids (CBC, CBD, CBDA, CBDV, CBG, CBGA, CBN, THC, Δ^8^-THC, THCA, THCV) at 25 µg/mL concentration was prepared in MeOH (HPLC grade) from a commercial 250 µg/mL solution (Cayman Chemical Company, Ann Arbor, MI, USA). Ethanol 200 proof anhydrous (Decon Laboratories Inc.) and HPLC grade methanol, certified ACS formic acid optima LC/MS grade and Ottawa sand (20 – 30 mesh) were purchased from Thermo Fisher Scientific (Waltham, MA, USA). Ultrapure water (18 MΩ-cm) was produced using a Barnstead NanoPure Infinity Ultrapure Water System (Thermo Fisher Scientific, Waltham, MA, USA). Ultra-high purity nitrogen was obtained from Airgas (Pueblo, CO, USA).

### Pressurized liquid extraction (PLE)

All kinetics experiments were carried out on Thermo Scientific™ Dionex™ ASE-350 pressurized liquid extractor. In order to increase the efficiency of extraction process, the ASE-350 combines conventional solvents with high temperatures and pressures. As a result, run times are shorter and the amount of solvent used is significantly less. The pressurized liquid extraction can be operated at a pressure of approximately 11.0 MPa and at a temperature range from ambient to 200 °C to efficiently extract analytes from the complex matrices. The methods and sequences were created and run, with the Chromeleon software, version 7.2 SR5 (Thermo Fisher Scientific, Waltham, MA, USA) on Microsoft Windows 8.1 operating system. For experiments, a 10 mL stainless steel extraction cell containing from bottom to top, a glass fiber filter (Thermo Fisher Scientific, Waltham, MA, USA), 1.5 g of Ottawa sand, ~ 1.0 g of homogenized dry hemp, and lastly to ensure regular solvent volumes, ~ 5–6 g Ottawa sand was again used to fill any residual space. The cell was then sealed and placed into the cell tray of the ASE-350.

A previously optimized method on CBDA, using PLE (Olejar and Kinney, 2021), which is a thermo-chemical conversion in water utilizing the parameters of 140 °C temperature and two static cycles of 3 min each, followed by extraction in ethanol at 120 °C temperature for two static cycles of 3 min each was utilized as the control. The chosen solvent for the decarboxylation was water due to the sparing solubility of cannabinoids in water. Two parameters were examined for the kinetics of decarboxylation process: extraction temperature and time, on hemp samples using the automated ASE-350. A total of nine time points (1, 2, 5, 10, 20, 30, 45, 60, 90 in minutes) at each thermo-chemical conversion temperature (80, 100, 120, 140 and 160 °C) in triplicates, were performed. Then extraction of the decarboxylated hemp was performed using ethanol at 120 °C and two static cycles for 3 min each. Each extracted volume in collection vials, was measured prior to filtering. The neat ethanol extract along with 1:100 and 1:10 dilutions in MeOH then underwent further analysis by reverse phase HPLC.

### High performance liquid chromatography (HPLC)

All Cannabinoids were quantified by liquid chromatography on a Thermo Scientific Dionex UltiMate 3000 HPLC system (Thermo Fisher Scientific, Waltham, MA, USA) equipped with a temperature controlled autosampler (WPS 3000TSL Analytical), a column oven compartment (TCC-3000SD) and a diode array detector with multiple wavelength detection (DAD 3000 and MWD 3000). The Chromeleon 7.2 software, version 7.2 SR5 (ThermoFisher Scientific, Waltham, MA, USA) was used in the system to control the measurements. Chromatographic separation of cannabinoids was accomplished using an Accucore aQ C18 Polar Endcapped column, I.D. 30 mm × 3 mm, particle size 2.6 μm (Thermo Fisher Scientific, Waltham, MA, USA) maintained at 25 °C. To achieve the effective separation of the cannabinoids a gradient was employed consisting of mobile phase A, 0.1% formic acid in water, mobile phase B, 0.1% formic acid in methanol. The gradient started at 62% B, increasing to 66% B at 13.75 min, followed by an increase to 80% B at 20 min. After maintaining it for 4 min, then returning to 62% B and equilibrating for 3 min, the total runtime was 24 min. A flow rate of 0.45 mL/min was utilized along with a 2.0 μL sample injection volume was used. Analyte presence was measured at the wavelengths of 210 nm and 220 nm. Cannabinoids in extracts were verified by comparison to the retention time and to the UV spectra of the pure cannabinoid standards. A four-point standard curve (5, 10, 50, 100 µg/mL) having linear regression (*r*^2^ > 0.99) was used to quantify the cannabinoids.

The system calibration was monitored using a commercially available standard solution consisting of 11 cannabinoids diluted to a concentration of 25 µg/mL (continuing calibration verification (CCV) standard). The CCV was analyzed following the standard curve and after every 10 samples injections. Analyses of samples were performed in duplicate to ensure the performance of the instrument and accuracy of the results.

## Results and discussion

### Decarboxylation studies

**Fig. 2:**
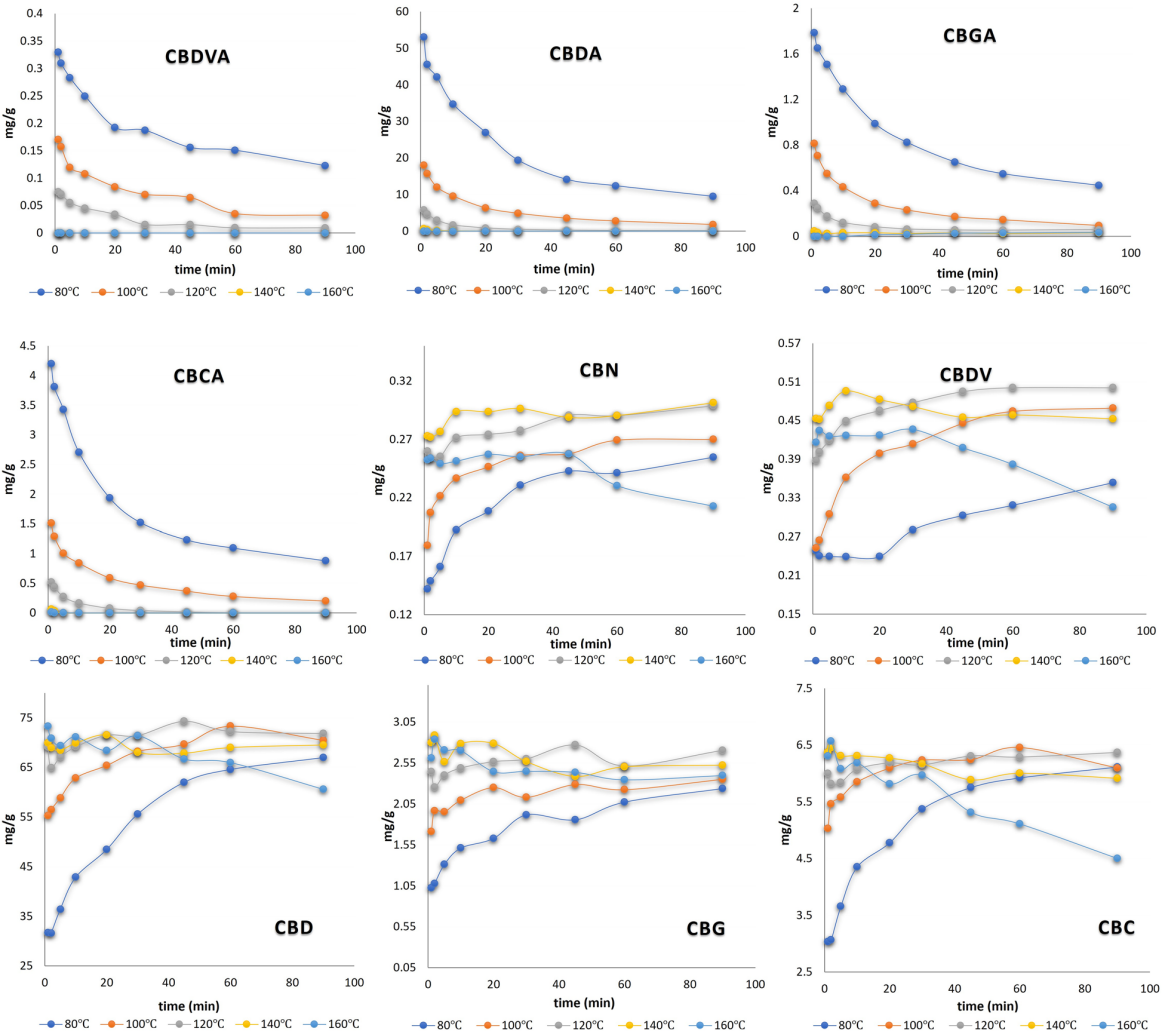
Cannabinoid concentrations in ethanolic extract as a function of time and temperatures

The concentrations of individual cannabinoids extracted from hemp were calculated over five different temperatures (80, 100, 120, 140, or 160 °C). For each temperature, a series of thermo-chemical conversions were performed over time points of 1, 3, 5, 10, 20, 40, 60, and 90 min. After experiments, the concentrations (mg/g) were plotted as a function of time (min) and temperature (°C) (Fig. 2). A relatively low conversion of all acidic cannabinoids into their respective neutral form was found at 80 °C, while an appreciably increase in conversion was shown in accordance with increasing temperature. However, at an elevated temperature of 160 °C the concentrations of all neutral forms started decreasing after 30 min of exposure likely resulting from degradation of the neutral cannabinoids. For instance, the CBGA to CBG and CBDA to CBD decarboxylation at 140 °C temperature, continued to show maximum concentration after 20 min. The CBDA to CBD decarboxylation was in good agreement with the earlier published report for this transformation (Olejar and Kinney, 2021). At a temperature of 140 °C CBD was maximum for over 20 min and almost remained constant throughout the 90 min, however at 160 °C the concentration of CBD started decreasing after 45 min. We predicted the reason for this to be the thermolability of cannabinoids resulting in unknown degradations products due to the increased temperature. Similar results were observed in the case of CBGA to CBG and CBCA to CBC. Interestingly, at elevated temperature (160 °C, or 140 °C in some) CBDA, CBGA and CBCA were completely decarboxylated in less than 1 min. This was attributed to the extraction cell requiring heating prior to the introduction of water (Fig. 2). At 140 °C, the maximum CBDV concentration resulted at 10 min. CBN accumulation was noted at higher temperatures, which is a well-known oxidation product of THC, but could not totally account for the observed losses of THC.

**Fig. 3:**
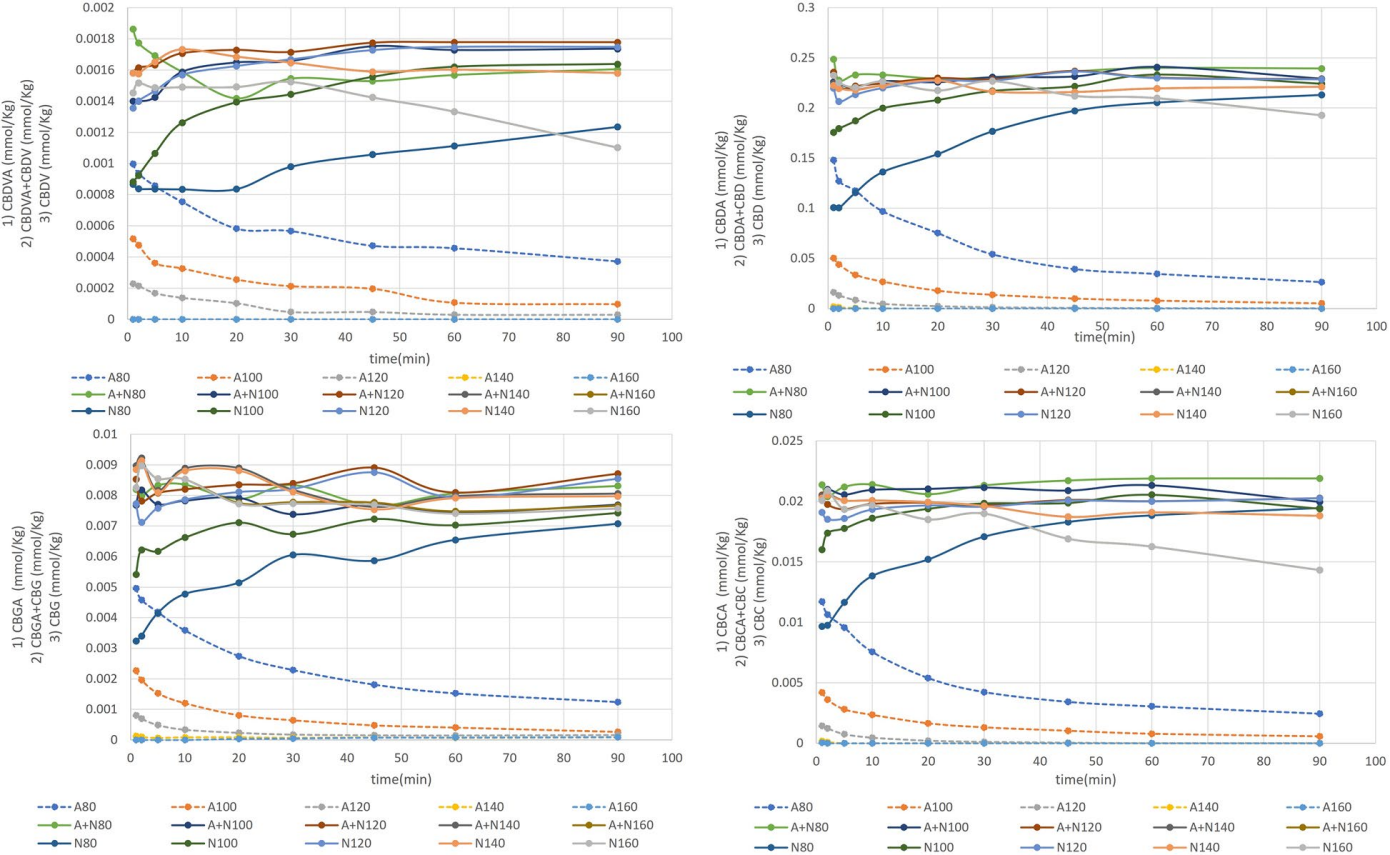
Cannabinoid molar concentration plots as a function of time and temperature. Dotted lines represent the acid cannabinoid molar concentrations while different solid color lines represent neutral and the combination of acid plus neutral cannabinoid molar concentrations

According to earlier literature the decrease in total cannabinoid concentration at elevated temperatures is a common observation. A loss of 60% in molar concentration of CBD_total_ at 120 °C, during hemp seed oil decarboxylation in open reactors was observed by Citti et al. (2018). Wang et al. has mentioned the unexplained decrease in THC, CBG and CBD forms in cannabis extracts when decarboxylation was carried out at 145 °C in a vacuum oven (Wang et al., 2016). Veress et al. (1990) also observed the same for CBD and THC at 122 and 145 °C attributing the losses to evaporation. According to a study of cannabis resin over several years under different storage conditions, the increase in total CBN concentration does not correspond to the decrease in total THCA + THC, explaining that degradation of THC can occur into other unknown compounds (Lindlost, 2010; Trofin et al., 2012). Another study on cannabis plant material under the effect of storage temperature disclosed that temperatures of 100 °C and above could lead to an accelerated THCA decarboxylation process followed by fast and rapid loss in THC. Likewise, Moreno et al., has also observed that the total molar concentration of CBD and CBG (sum of their acidic and neutral forms) plummeted by 90%, after 60 min. at 160 °C (Moreno et al., 2020). It was predicted to be the result of unidentified side products formation along with evaporative losses at higher temperature since the boiling point of CBG, THC and CBD lie in the range of 120—180 °C. During the kinetics analysis of thermo-chemical conversion in PLE, such losses in concentrations turned out to be insignificant. The sigmoidal curves for the molar concentrations of acid and neutral cannabinoid, came out equal and opposite as illustrated (Fig. 3). This fact was further confirmed when the combination of acid and neutral cannabinoid molar concentrations were plotted, and the result was almost a linear line through the time period, ruling out any possibility of a rise or drop in total concentration.

### Kinetic Model

**Scheme 1:**
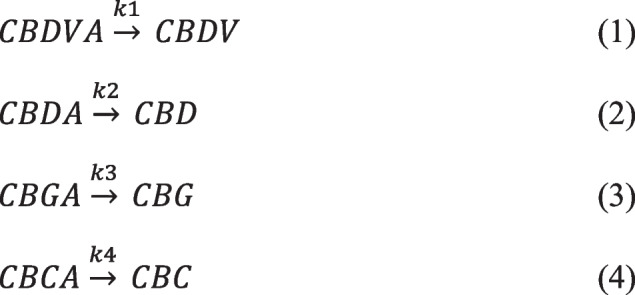
Individual cannabinoid decarboxylation reaction matrices

To understand the kinetics of the thermo-chemical conversion of cannabinoid acids to neutral cannabinoids a series of experiments examining cannabinoid contents following the conversion process were undertaken. Each series of experiments involved examining the concentration of the cannabinoid and its corresponding acid over a time range at a selected temperature. Utilizing this data, the reaction order was established from the graphical plots of this data. Considering the reaction matrix or simple model for CBDVA, CBDA, CBGA, and CBCA decarboxylation given in Scheme 1, where *k*’s are the rate constant for that cannabinoid decarboxylation.

The reaction matrix (Scheme 1) can be simplified into the Eqs. (1–4) given below.

1 $$\frac{d[CBDVA]}{dt}=k1[CBDV]$$

2 $$\frac{d[CBDA]}{dt}=k2[CBD]$$

3 $$\frac{d[CBGA]}{dt}=k3[CBG]$$

4 $$\frac{d[CBCA]}{dt}=k4[CBC]$$

**Fig. 4:**
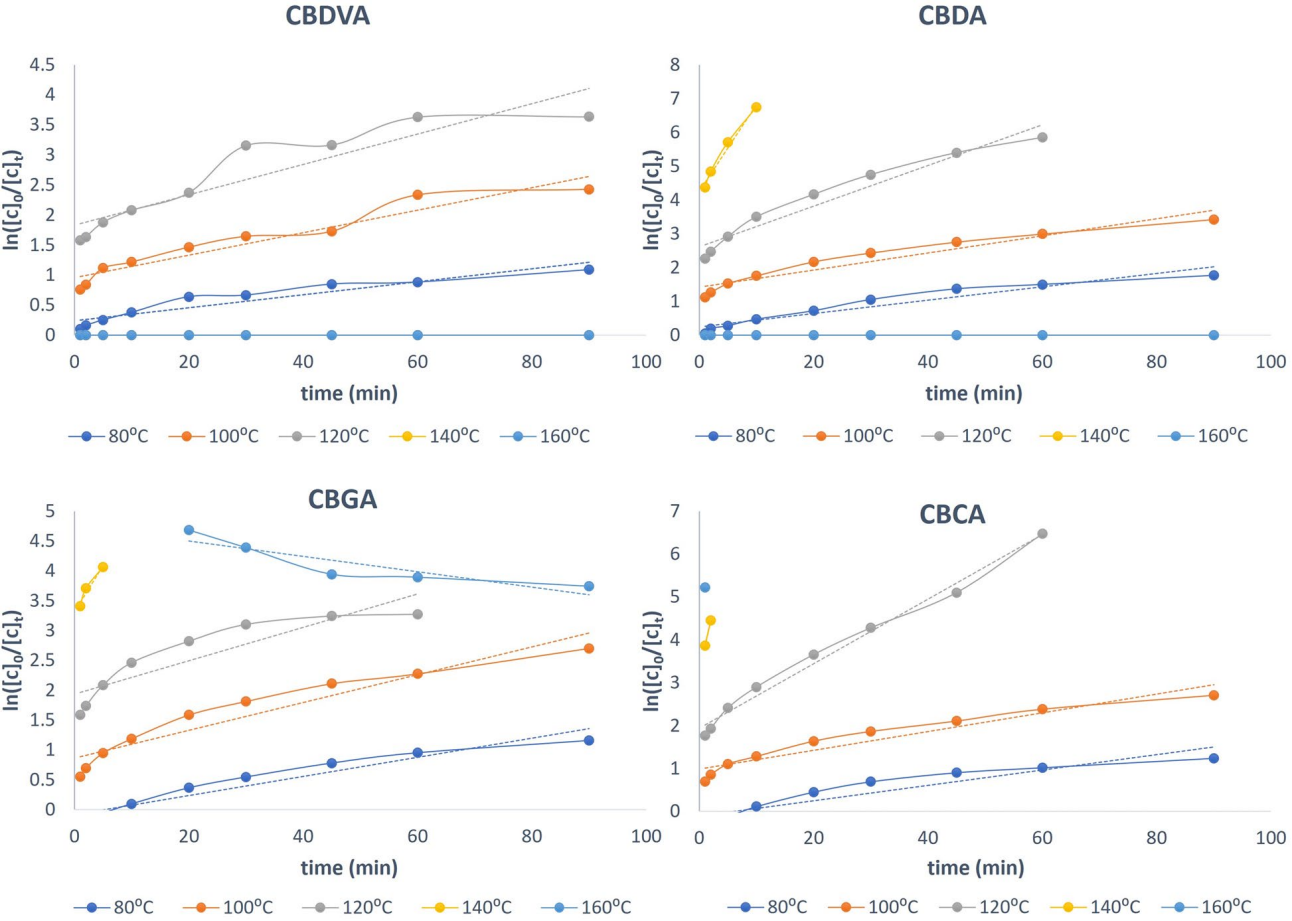
Thermo-Chemical decarboxylation kinetics at different temperatures

Upon integration, the above Eqs. (1–4) can be converted into the generalized Eq. 5

5 $$ln\frac{[Co]}{[Ct]}=kt$$

where [C_0_] and [C_t_] stand for the acidic cannabinoid concentration at time 0 and t minutes, respectively. The concentrations following extraction on non-decarboxylated hemp exclusively are shown by the symbol [C_0_] i.e. when decarboxylation time is zero. The rate order was established by plotting $ln\frac{[Co]}{[Ct]}$ vs time (Fig. 4). Each plot was examined for its linearity. A linear line was obtained from the plot of these values and from the resulting lines equations the value of *k* is determined. The graphical representation of the data is displayed (Fig. 4) and the extracted linear regressions and equations of best-fit line are expressed (Table 1). It should be noted from this data that at the higher temperatures 160 °C and occasionally 140 °C the reaction goes to completion almost immediately. In instances when a representative line, of at least three data points, is not available the temperature was excluded from further calculations. The reaction order was found to be a pseudo-first order reaction and the rate constant, *k*, was found to be equal to the slope of this line. A pseudo-first order reaction is defined as a reaction that appears to be first order: however, one reactant is typically found in gross excess so its change in concentration is negligible, or one reactant is a catalyst. Since thermo-chemical conversion utilizes water in excess compared to cannabinoid content therefore the reaction is considered a pseudo-first order reaction.

**Table 1:** Cannabinoid reaction order determination, providing the coefficient of correlation and equation of the best-fit line

Temperature	Coefficient of correlation (*R*^2^)	Line equation
1) CBDVA		
80 °C	0.8880	y = 0.0108x + 0.2422
100 °C	0.9188	y = 0.0187x + 0.9568
120 °C	0.8551	y = 0.0253x + 1.8324
2) CBDA		
80 °C	0.9297	y = 0.0197x + 0.2477
100 °C	0.9237	y = 0.0253x + 1.4222
120 °C	0.9449	y = 0.0601x + 2.6145
140 °C	0.9814	y = 0.2556x + 4.2624
3) CBGA		
80 °C	0.9301	y = 0.016x – 0.0807
100 °C	0.9101	y = 0.0233x + 0.8626
120 °C	0.8118	y = 0.028x + 1.9348
140 °C	0.9463	y = 0.1536x + 3.3199
4) CBCA		
80 °C	0.8828	y = 0.0179x—0.1068
100 °C	0.9187	y = 0.0218x + 0.9882
120 °C	0.9873	y = 0.0753x + 1.9357
140 °C	1.000	y = 0.5896x + 3.2733

At the higher temperatures 160 °C and occasionally the140 °C when the reactions have completed almost immediately, a representative line, three data points, is not available, the temperature is excluded from further calculations

**Fig. 5:**
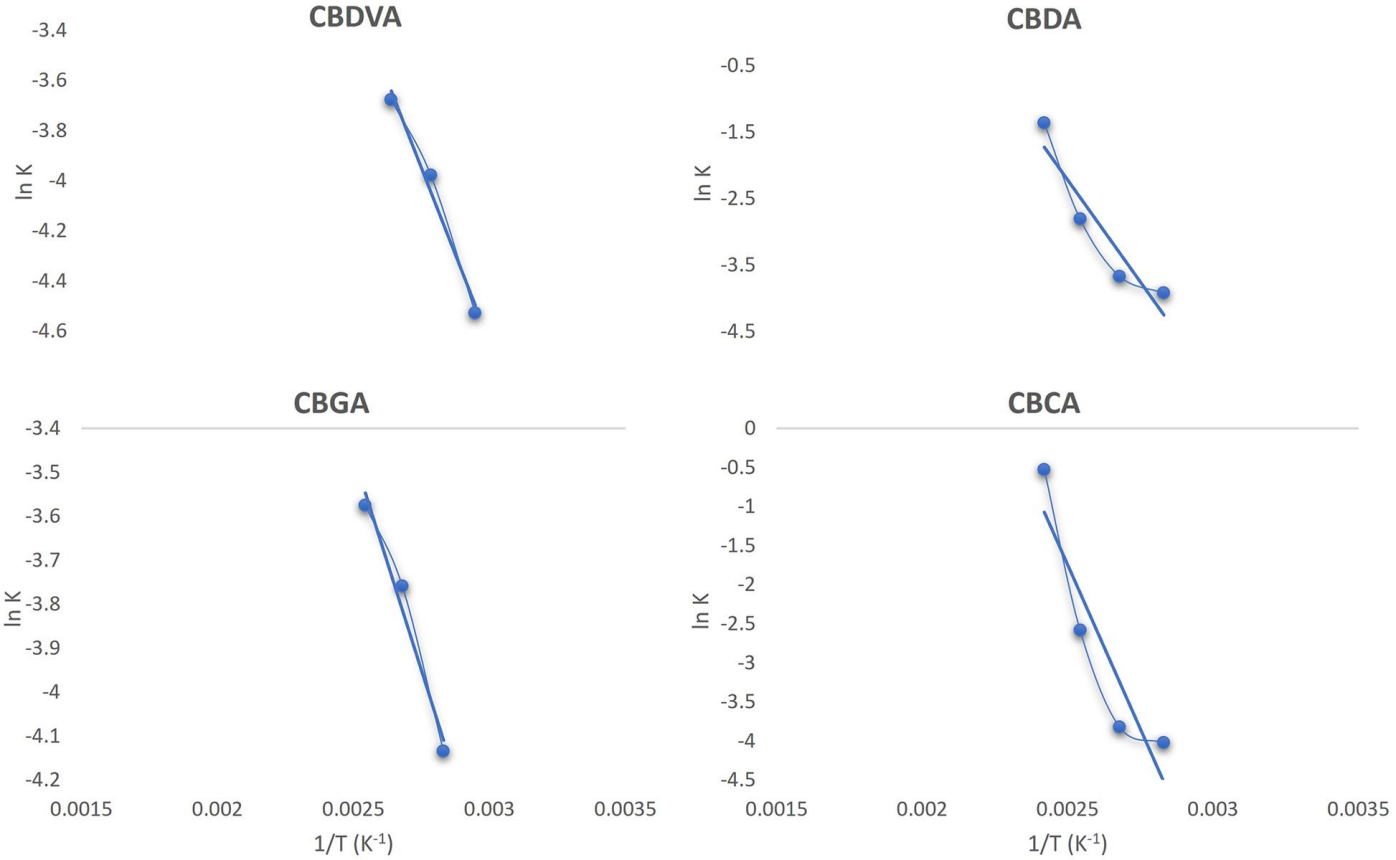
Graphical representation of the Arrhenius equations for cannabidivarinic acid (CBDVA), cannabidiolic acid (CBDA), cannabigerolic acid (CBGA), and cannabichromenic acid (CBCA)

This result was not unexpected as initially thermal decarboxylation was considered first order (Veress et al., 1990). Later it was discovered that this reaction was catalyzed by formic acid and it is now considered a pseudo-first order reaction (Perrotin-Brunel et al., 2011). However, in both these instances there is a large degree of loss, which results from evaporation of the cannabinoids and creates a complex kinetics model where degradation of the neutral cannabinoids must also be factored in with the evaporation of the cannabinoids. Furthermore, the value of 1/T, where T is the temperature expressed in °K, and ln(*k*) can be established. Once established these values may be plotted as ln(*k*) versus (1/T) following the Arrhenius equation to calculate the activation energy, *E*_*a*_, and the frequency or pre-exponential factor, *A* (Fig. 5). The value of the frequency constant, *A,* was established by obtaining the value of y when *x* = 0 using Eq. 6 and Eq. 7.

6 $$y=mx+c$$

where *y* = ln(*k*), m is the slope,* x* = (1/T), and c is the x-intercept.

7 $$\text{ln}k=\text{ln}A-\frac{{E}_{a}}{RT}$$

where *k* is the reaction rate constant, *A* is the frequency factor, *E*_*a*_ is the activation energy, *T* is temperature in °K, and *R* is the universal gas constant (8.3144 J K^−1^ mol^−1^). The obtained value of the x-intercept is the ln(*A*) and therefore Eq. 8 must be applied.

8 $$A={e}^{lnA}$$

The slope of the line is equal to ($\frac{-{E}_{a}}{R}$), where *R* is the gas constant and therefore must be converted to obtain the activation energy *E*_*a*_. The calculated rate constants, activation energies and frequency constant are included (Table 2).

**Table 2:** Rate constants, activation energies and pre-exponential factors for all acidic cannabinoids studied

	**(min**^**−1**^**)**	**80** °C	**100** °C	**120** °C	**140** °C	**Ea** **(kJ/mol)**	**A X 10**^**5**^** (min**^**−1**^**)**	**R**^**2**^
CBDVA	k_1_	0.0108	0.0187	0.0253	0.000	24.669	4.97E-04	0.9819
CBDA	k_2_	0.0197	0.0253	0.0601	0.256	51.039	5.04E + 00	0.8824
CBGA	k_3_	0.0160	0.0233	0.0280	1.54E-02	16.233	4.13E-05	0.9731
CBCA	k_4_	0.0179	0.0218	0.0753	0.590	69.809	2284.9026	0.8567

**Scheme 2:**
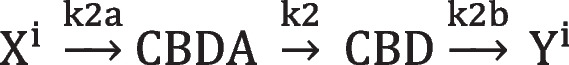
Reaction matrix of CBDA to CBD including unknown precursors and unknown degradation products

Expanding beyond this it is possible to calculate a mass balance corrected model Scheme 2).

Where *X*^*i*^ is an unknown precursor, *Y*^*i*^ is an unknown degradation product, and *k* are reaction rate constants.

Once the *k*, *E*_*a*_ and *A* values are established, using Scheme 1, a simple model of thermo-chemical conversion was generated. Unfortunately, this model does not consider other factors that contribute to the formation of neutral cannabinoids or degradation.

As such, these equations must be applied to obtain values of *k* for Scheme 2. From Scheme 2, a more complete model of thermo-chemical conversion was examined. Through this process *k*_2a_ and *k*_2b_ were found to negligible in the cannabinoids studied at the temperatures and times where maximum extraction was expected to occur. Consequently, the simple model was used for the predictive modeling of the cannabinoid thermo-chemical conversion and extraction.

**Fig. 6:**
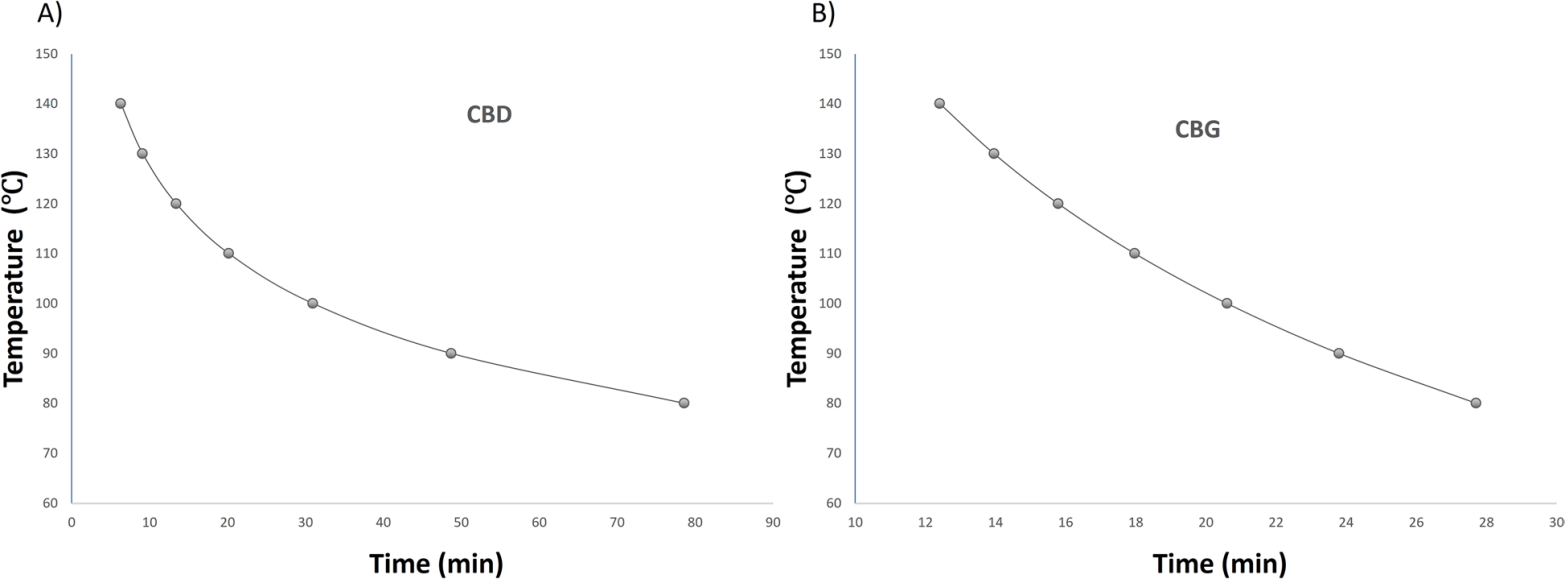
Prediction of the time required to reach the maximum cannabinoid concentrations and thermo-chemical conversion at different temperatures based on the simple model, **A**) CBD, **B**) CBG. Each time point in graphs, focusing on the maximum concentration of CBD (48.69 mg/g) and CBG (1.24 mg/g) respectively

The simple generated model further determined the favorable conditions leading to the maximum concentration of the desirable cannabinoid. The maximum concentration of CBDV, CBD, CBG, and CBC according to the model can be attained at each temperature, along with the predicted time (Fig. 6). The prediction graphs suggest that the thermo-chemical conversions using PLE are faster and take less time as compared to oven decarboxylation as reported by Moreno and others (Moreno et al., 2020). For instance, in the case of CBDA at 140 °C, the maximum concentration can be achieved in 6.3 min through thermo-chemical conversion, while thermal decarboxylation required 27 min at same temperature, based on the mass balance model of Moreno et al. (2020). According to the mass balance model of Moreno, the optimum conditions to get the maximum CBD is preferably at lower temperature (80 °C) with a longer time period (25 h), since at lower temperature the decomposition reaction is minimized for the associated higher activation energy of CBDA-CBD decarboxylation. However, in industry decarboxylation and isolation time are critical not only for being able to produce product but also to minimize operating expenses, such as power and labor. Consequently, thermo-chemical decarboxylation can be far superior.

### Prediction Model Verification

After the model was generated, it was verified for CBD and CBG maximum extraction concentration at a given temperature in two cannabis varieties. These two cannabinoids were chosen due to existing research and economic interests. Because the bench-top PLE does not perform fractions of a minute, times were rounded to the closest whole minute. The selected conditions for CBD were thermo-chemical conversion at 130 °C for 9 min using water as the solvent, followed by extraction with ethanol at 120 °C, for 2 static cycles of 3 min each. Similarly, the conditions for CBG were thermo-chemical conversion at 130 °C for 14 min followed by ethanol extraction at 120 °C for 2 static cycles of 3 min each.

The comparison of the means of triplicate processes of the control conditions and the model-generated conditions were tabulated (Table 3). It should be noted that while the model performs well for cannabinoids that are in larger quantity, it begins to falter when these compounds are in trace amounts. This is evidenced by the low predictions for the CBG in CBD rich hemp and the negative concentration of the CBD in CBG rich hemp. The reason for both errors is that the model's rate constants for the specified temperature and reaction time are higher than the cannabinoids concentrations present in the hemp. As a result, in both cases (CBD and CBG rich hemp), the extracted cannabinoid from the control and the model conditions, exceed the prediction.

**Table 3:** CBD and CBG model verification using CBD and CBG rich hemp varieties

	**CBD rich hemp (mg/g hemp)**	**CBG rich hemp (mg/g hemp)**
Model prediction CBD	62.8	-2.73
CBD control conditions	65.8 ± 3.6	0.299 ± 0.046
CBD model conditions	62.5 ± 2.4	0.213 ± 0.016
Model prediction CBG	0.82	46.8
CBG control conditions	2.40 ± 0.27	48.4 ± 9.5
CBG model conditions	2.98 ± 0.08	58.1 ± 5.3

The models performed as expected for the major cannabinoids in each variety. The CBD model predicts the CBD values for the control, which the model was based on, and the model conditions. While the CBG model predicts the values for the control well, the model conditions outperform the prediction. This discrepancy may be due to the model being generated from a variety of hemp that is not high in CBG or there may be an unknown precursor forming CBG under the predicted conditions. The latter was not expected, as there was no evidence of a precursor during the model development. Furthermore, it is not expected that the hemp variety used will affect the kinetics of the process; however the kinetics of the cannabinoids in the associated cannabinoid-rich matrix may differ based on concentration. Consequently, further studies should be done to elucidate the relationship of cannabinoid concentration to *k* values.

## Conclusion

Thermo-chemical conversion for decarboxylation has exciting new possibilities for the decarboxylation of acidic cannabinoids found in cannabis. To fully understand the process, the reaction kinetics was examined. It should be mentioned that each experiment was carried out utilizing a consistent volume and diameter of cell and a controlled amount of hemp. Time and temperature are taken as a variable in this case, which does not rule out the influence of hemp loading.

The thermo-chemical conversion of cannabinoids follows pseudo-first order kinetics. The order of activation energies to convert from the acidic form to the neutral form were found to be CBCA > CBDA > CBDVA > CBGA. Utilizing the kinetics data and calculations, it was possible to generate a simple model for the prediction of extraction concentration as well as to examine the possibility of a complex model taking into account unknown sources of neutral cannabinoids and degradation products. It was established that the simple model best represented the thermo-chemical conversion process as unknown sources and degradation products were negligible. Furthermore, application of the model to two varieties of cannabis demonstrated its efficacy in predicting maximal extraction concentrations in the corresponding cannabinoid rich environment. This understanding will prove to be beneficial in the up scaling of the process from bench top to pilot and commercial scale. Lastly, further work is required to understand the dynamics of the model for predicting the extraction of minor cannabinoids.

## Data Availability

The data will be made available upon reasonable request.

## Abbreviations

EV: Extracellular vesicles
A: Frequency Factor
CBC: Cannabichromene
CBCA: Cannabichromenic acid
CBD: Cannabidiol
CBDA: Cannabidiolic acid
CBD_total_: Total Cannabidiol
CBDV: Cannabidarin
CBDVA: Cannabidivarnic acid
CBG: Cannabigerol
CBGA: Cannabigeric acid
CBN: Cannabinol
CCV: Continuing Calibration Verification
cGMP: Current Good Manufacturing Procedures
DAD: Diode Array Detector
*Ea*: Activation Energy
HPLC: High Performance Liquid Chromatograpy
ECS: Endocannabinoid System
k: Reaction Rate Constant
MeOH: Methanol
MWD: Multiple Wavelength Detector
PPcP: Pharmaceutical and Personal Care Products
PLE: Pressurized Liquid Extraction
PWHE: Pressurized Hot Water Extraction
THC: Δ^9^-Tetrahydrocannabinol
THC_total_: Total Δ^9^-Tetrahydrocannabinol
THCA: Δ^9^-tetrahydrocannabinolic acid
THCV: Δ^9^-tetrahydrocannabivarin
THCVA: Δ^9^-tetrahydrocannabivarinic acid
Δ^8^-THC: Δ^8^- tetrahydrocannabinol
